# Practices and challenges of infectious waste management: A qualitative descriptive study from tertiary care hospitals in Pakistan

**DOI:** 10.12669/pjms.314.7988

**Published:** 2015

**Authors:** Ramesh Kumar, Babar Tasneem Shaikh, Ratana Somrongthong, Robert S Chapman

**Affiliations:** 1 Ramesh Kumar, PhD. Assistant Professor, Department of Health Systems and Policy, Health Services Academy, Islamabad, Pakistan. College of Public Health Sciences, Chulalongkorn University, Bangkok, Thailand; 2 Babar Tasneem Shaikh, PhD., FRCP, Associate Professor, Department of Health Systems and Policy, Health Services Academy, Islamabad, Pakistan; 3 RatanaSomrongthong, PhD Associate Professor, College of Public Health Sciences, Chulalongkorn University, Bangkok, Thailand; 4 Robert S Chapman, MD Faculty, College of Public Health Sciences, Chulalongkorn University, Bangkok, Thailand

**Keywords:** Infectious waste management, Tertiary care hospitals, Healthcare personnel, Pakistan

## Abstract

**Background and Objective:**

Infectious waste management practices among health care workers in the tertiary care hospitals have been questionable. The study intended to identify issues that impede a proper infectious waste management.

**Methods:**

Besides direct observation, in-depths interviews were conducted with the hospital administrators and senior management involved in healthcare waste management during March 2014. We looked at the processes related to segregation, collection, storage and disposal of hospital waste, and identified variety of issues in all the steps.

**Results:**

Serious gaps and deficiencies were observed related to segregation, collection, storage and disposal of the hospital wastes, hence proving to be hazardous to the patients as well as the visitors. Poor safety, insufficient budget, lack of trainings, weak monitoring and supervision, and poor coordination has eventually resulted in improper waste management in the tertiary hospitals of Rawalpindi.

**Conclusion:**

Study has concluded that the poor resources and lack of healthcare worker’s training in infectious waste results in poor waste management at hospitals.

## INTRODUCTION

Infectious waste is produced from the hospitals during the diagnosis, immunization, surgical procedures and treatment of patients, and can transmit the infections to the hospital staff, attendants, and the nearby public.^1^ Infectious waste comprises 10-25% of all the waste produced in hospital, which cannot be disposed off with the normal domestic waste. However, this is a common observation in many hospitals of the developing countries. Infectious waste includes the body fluids or secretions (e.g., blood, pleural fluid, semen, vaginal secretions, vomit, feces or urine), contaminated sharp objects (e.g., contaminated needles, syringes and surgical blades), biological laboratory waste (e.g., cultures, stocks and growth media), pathological waste (such as human tissue, organs or body fluids), and single-use disposable equipment, utensils and instruments soiled with potentially infectious agents. Infectious waste generation rates, normally depends on the size of hospital, number of patients coming to that particular facility, number of beds available, segregation steps and kind of care provided to the patients.

Studies proved around 1.35 Kg / bed healthcare waste has been generated by the tertiary care hospital of Pakistan. About 92,000 beds are available only at the tertiary care hospitals of public sector in Pakistan that produces 0.8 million tons of waste every day.^2^ Infectious waste management is a big challenge for hospital administration in limited resource settings and Pakistan is not an exception.^3^ Rapid population growth, patient load on hospitals and negligible investment in healthcare waste management measures have posed a serious public health hazard and threat.

Insufficient training of health workers results inimproper infectious waste handling and disposal.^4^ Infectious waste is handled in four steps: segregation, collection and transportation, storage and disposal. This waste must be treated prior to its final disposal by the autoclave or by incineration.^4^ Most healthcare workers do not follow the proper waste management guidelines and encounter sharp injuries and infection.^5^

One of the WHO study revealed that two thirds of hospitals among 22 countries were not following the proper infectious waste management practices.^6^ Therefore, a continuous training on infectious waste was suggested for healthcare workers (HCWs) to control the menace of infectious diseases that can potentially endanger the patients, attendants, hospital staff, and residents in the neighborhood.^7^,^8^

This study endeavored to describe the situation of infectious waste management practices among health care workers working at the Holy Family Hospital and the District Headquarter Hospital of Rawalpindi Pakistan. The study also identified the hidden issues, and barriers responsible for poor infectious waste handling in the hospitals.

## METHODS

It was a qualitative descriptive study with cross sectional design. Two qualitative approaches; direct observation and in-depth interviews were conducted during March 2014. Direct observation and physical verification was carried out, using validated WHO checklist for segregation, collection, storage and disposal of infectious waste.^9^ All departments of the hospitals were included. In addition, in-depth interviews were conducted till the point of saturation. Principal investigator himself conducted ten in-depth interviews, using WHO semi-structured questionnaire, after taking the appointment and written consent. Respondents included the Medical Superintendent, Executive Director, Deputy Director, Nursing superintendent and a focal medical officer, dealing with the waste management. Verbatim notes were taken and interviews were recorded, with permission.

Data collected was transcribed and a thematic content analysis was done. Specific nodes were developed for the questions, and significant findings and responses were aggregated as sub-nodes, which were later developed into themes. Information from literature and responses were then triangulated in the discussion section.

### Ethical consideration

Ethics approval for this study was granted by the Institutional Review Board of Health Services Academy, Islamabad-Pakistan. Verbal informed consent was obtained from all the administration staff of both hospitals, after explaining the objectives of the study. Confidentiality and anonymity was assured to all the participants. Data was kept under lock and key with the principal researcher.

## RESULTS

### Direct observations

We closely observed four recommended steps of infectious waste management in both the hospitals:

### Segregation

Each department has four color coded waste bins: red for infectious, black for general, yellow for sharps and white safety box for injection safety; however there wasn’t any proper labeling on the bins. Three departments from control and two from intervention hospitals were using only two bins of different colors. There were no separate bins for the hazardous waste such as pharmaceutical waste, chemical waste and the radioactive waste. They were using either red or yellow bin for these kinds of wastes. Black waste bin was found at the patient’s bed side, and was being used for all sorts of waste. Red bin with infection safety box was although placed at the nursing station, yet it was uncovered. The HCWs were not segregating the infectious waste, as some of the patient’s blood stained objects were seen in the general waste bin.

### Collection

Waste is collected and transported thrice a day by the sanitary workers in a simple uncovered trolley. Trolleys with infectious and the non-infectious waste together were driven through the common passages in both the hospitals, and were not even washed afterwards. General waste included common papers, used plastics bags, hard papers and files, food boxes, kitchen items, fruits waste etc. Plastic collection bags were not properly sealed and some of them were not even intact because of being filled beyond their capacity. At three places, only infectious waste was being collected, and that too without labeling. The sanitary workers did not use personal protective equipment (PPE) such as gloves, long rubber boots, aprons and masks during waste collection. Hence, WHO guidelines were not followed at all.

### Storage

There are separate storage points located in both the hospitals. General waste was dumped in an open container, which is daily emptied by the municipality for disposal. Nevertheless, used syringes, blood drip sets, medicines vials and urine bags were also found inside general waste containers. There is a storage room for the infectious waste with no temperature control system for pathological wastes. The HCWs were again found to be violating the guidelines for the storage of waste. The capacity of storage areas in both hospitals was not enough to hold the quantity of infectious waste produced every day.

### Disposal

In both hospitals, the autoclave and three chamber incinerators were used for the final disposal of the infectious waste. However, there is no back up for both the machines. Incinerators were installed away from main building, and were fairly well-maintained. Local municipality uses land filling for the disposal of general waste. Again PPE were not used during the disposal.

### Findings from in-depth interviews

Respondents included 4 female and 6 male staff, regular government employees who were overseeing the management of infectious wasteas Medical Superintendent, Executive Director, Deputy Director, Nursing superintendent and a focal medical officer. Thematic results are presented here.

### Poor safety of the workers

Respondents at both hospitals agreed that the PPE is not available for quite some time. Therefore, the workers are at great risk during waste handling. The Deputy Director of one hospital said:

*“Needle prick injuries are the most common hazard during the infectious waste management at our hospital”*.

### Insufficient Budgeting

The respondents, especially the nursing staff admitted that there should be adequate budget for equipment used in infectious waste management. The hospital management admitted that the facility doesn’t have color coded waste bins for infectious waste, and it results in mixing with non-infectious waste. The Executive Director from one hospital said:

*“Most of the time, we don’t have the enough funds for purchasing the PPE and waste bins”*.

### Lack of trainings

All the respondents demanded that thereshould be a regular training on infectious waste management in both hospitals for all cadres of HCWs. They believe that training would surely change the practices of staff by increasing their knowledge on infectious waste**, and that without training, workers cannot perform in an efficient way**. The Medical Superintendent from one hospital said:

*“Training would be more beneficial for improving waste management practices at the hospital, if conducted on regular intervals and for every new batch of health personnel (doctors, nurses, paramedics, auxiliary staff etc.) inducted in the hospital”*.

### Weak supervision and monitoring

All the respondents were of the opinion that the supervision of HCWS involved in waste management is extremely important, so that they perform their job diligently. The MS from one hospital said:

“*I am very much concerned about the regular monitoring & supervision during the working hours, & I have directed the relevant deputy MS to comply with the standards”*.

### Poor coordination

Almost everybody felt the need for regular meetings for improving the management of infectious hospital waste**. The Deputy Director from the control District headquarter hospital Rawalpindi said,

*“Regular meetings result in better coordination between various departments, and we can work better toward infectious waste management*”.

The Medical Superintendent from one hospital shared his experience of such meetings for improving the knowledge of the staff at the hospital.

## DISCUSSION

This is the first study of its kind in Pakistan’s public sector health care system, which documented that WHO guidelines for the infectious waste management are not being followed. This major finding is consistent with another study conducted in similar setting.^8^ The need for regular training for building the capacity of hospital workers in infectious waste management has been alluded to, and confirms our observation too in both the hospitals. Other researches have also recommended that such training are critical for improving the practices of health workers.^2^,^9^ Various issues pertaining to segregation of waste at point source, inappropriate collection, transportation, storage place, disposal and lack of PPE were marked first through direct observations, and later mentioned by the respondents. Hospital waste management requires not only committed and skilled workers, but their close monitoring as well.^10^ Every hospital must keep the environment free from infection.^11^ Therefore, a periodic evaluation on the disposal methods in hospital would be imperative.^12^

As explained by the respondents lack of funds for training for the staff reflects that waste management is a neglected issue, and that authorities perhaps are not sensitized enough on the magnitude of this problem. The non-availability of the waste bins and PPE is the main reason behind the inappropriate practices at the hospitals. It is known that the healthcare workers are the high risk groups in hepatitis B and C infections at the hospitals due to frequent needle prick injuries.^13^,^14^ The HCWs are often unaware about the consequences of poor waste segregation, and that training does improve their knowledge, practices and efficiency about waste management, besides building their confidence.^15^,^16^ Moreover, continuing capacity building of the staff and inculcating attitudinal change can ensure their safety too.^17^ Proper allocation of budget and arrangement of infectious waste management training at any hospital should be spelled out in health policy.^18^

A continuous supervision and monitoring could increase the motivation of health staff and ultimately affects their better working output on time.^19^,^20^ Regular watch on the health care workers has actually brought good results and improvement in the infectious waste management in the hospital.^21^ Hospital should ensure the implementation of waste management plan to avoid the health and environmental hazards.^22^,^23^

## CONCLUSION

A continuous and a comprehensive training of health personnel in various cadres could improve the infectious waste management practices in the hospitals. However, a waste management plan, appropriate equipment, dedicated staff, and robust monitoring and supervision are some of the pre-requisites. Nonetheless, hospital administration’s will is the foremost driver to bring about the change. More such studies could guide the interventions for improvement in the management of hazardous waste in the hospitals.
